# Comparative serum proteomics of plasmodium-infected free-living macaques in Thailand: Host immune responses and candidate biomarkers for zoonotic malaria

**DOI:** 10.14202/vetworld.2025.3051-3069

**Published:** 2025-10-20

**Authors:** Pakorn Ruengket, Sittiruk Roytrakul, Daraka Tongthainan, Chanya Udom, Wirasak Fungfuang

**Affiliations:** 1 Genetic Engineering and Bioinformatics Program, Graduate School, Kasetsart University, Bangkok, Thailand; 2 Functional Proteomics Technology Laboratory, National Center for Genetic Engineering and Biotechnology (BIOTEC), National Science and Technology Development Agency, Pathum Thani, Thailand; 3 Faculty of Veterinary Medicine, Rajamangala University of Technology, Tawan-ok, Chonburi, Thailand; 4 Department of Zoology, Faculty of Science, Kasetsart University, Bangkok, Thailand

**Keywords:** biomarkers, host–pathogen interactions, macaques, *Plasmodium*, proteomics, zoonotic malaria

## Abstract

**Background and Aim:**

Zoonotic malaria poses an emerging challenge in Southeast Asia, where rapid urbanization and deforestation increase human–wildlife interactions. Macaques are important natural reservoirs for *Plasmodium* species due to their evolutionary proximity to humans. Understanding host-specific immune responses to malaria in free-living macaques may aid biomarker discovery and guide surveillance strategies. This study aimed to characterize serum proteomic profiles in three wild macaque species, *Macaca fascicularis* (Mf), *Macaca leonina* (Ml), and *Macaca arctoides* (Ma), naturally infected with zoonotic *Plasmodium* spp., and to identify conserved proteins with biomarker potential.

**Materials and Methods:**

Blood samples from 61 macaques (25 Ml, 32 Ma, 4 Mf) were screened for *Plasmodium* species using nested polymerase chain reaction (PCR). Serum proteomes were analyzed using liquid chromatography-tandem mass spectrometry, followed by bioinformatics-based differential protein expression analysis, pathway enrichment, gene ontology classification, and construction of protein–protein interaction (PPI) networks. Comparative analyses were performed across species to identify conserved expression signatures.

**Results:**

Nested PCR confirmed *Plasmodium* infections in 20.00% of Ml, 50.00% of Ma (34.78% zoonotic), and 100% of Mf samples. Proteomic profiling identified 9,525 serum proteins, of which 698 were differentially expressed across species. Thirty-six proteins formed robust PPI subnetworks linked to immune defense, apoptosis, DNA repair, calcium signaling, and cytoskeletal remodeling. Ml exhibited predominant protein upregulation, whereas Mf and Ma showed downregulation trends, indicating species-specific immune adaptations. Notably, six of nine previously reported malaria-associated proteins (including CARD domain-containing protein, interleukin 1 receptor-associated kinase 1, and non-specific serine/threonine protein kinase) were consistently expressed across all species, supporting their potential as conserved biomarkers.

**Conclusion:**

Free-living macaques demonstrate distinct proteomic responses to *Plasmodium* infection, with Ml mounting a stronger immune response relative to Ma and Mf. The identification of conserved immune-related proteins highlights their translational potential as biomarkers for zoonotic malaria in humans. These findings advance the understanding of host–parasite interactions in natural macaque populations and provide a foundation for selecting optimal primate models, improving surveillance, and developing targeted interventions against zoonotic malaria.

## INTRODUCTION

Malaria continues to be a major global public health challenge, particularly in tropical and subtropical regions. The disease is caused by protozoan parasites of the genus *Plasmodium* and transmitted to humans primarily through the bites of infected female *Anopheles* mosquitoes [1]. According to the World Health Organization (WHO), approximately 263 million malaria cases were reported globally, representing an increase of 11 million compared with the previous year. The African region bears the heaviest burden, accounting for nearly 94% of cases, although malaria-related mortality has declined overall [2]. Among the human-infecting parasites, *Plasmodium falciparum* is the leading cause of severe morbidity and mortality, whereas *Plasmodium vivax*, though less lethal, is widespread and capable of causing severe disease [3, 4].

Significant progress in malaria control has been achieved through the development of antimalarial drugs, vaccines, and large-scale intervention programs [5]. The WHO has set an ambitious target to eliminate malaria worldwide by 2030 [6]. Beyond humans, more than 250 *Plasmodium* species have been identified across mammals, birds, reptiles, bats, and non-human primates (NHPs) [7, 8]. Six species are known to naturally infect humans: *P. falciparum, P. vivax, Plasmodium malariae, Plasmodium ovale curtisi, Plasmodium ovalewallikeri*, and *Plasmodium knowlesi* [9]. Increasing evidence also highlights the zoonotic potential of simian malaria parasites, including *Plasmodium cynomolgi, Plasmodium inui, Plasmodium fieldi, Plasmodium simium*, and *Plasmodium brasilianum* [10]. Of these, *P. cynomolgi* has gained prominence in Asia due to its growing risk profile, now considered comparable to that of *P. knowlesi* [11]. Such parasites can infect humans naturally or experimentally, underscoring the underestimated risk of zoonotic transmission [12]. For example, zoonotic malaria cases in Thailand rose from 176 in 2022 to 195 in 2023 [13], while Malaysia reported 2,505 cases and nine deaths in 2023, hindering progress toward malaria-free certification [9]. These trends are exacerbated by deforestation and urban expansion, which intensify human–wildlife interactions and the risk of cross-species transmission [14, 15].

The evolutionary closeness between humans and NHPs, coupled with shared mosquito vectors, facilitates malaria transmission across species [16]. NHPs are therefore not only natural reservoirs but also valuable models for zoonotic disease research due to their genetic, immunological, and physiological similarities to humans [17]. Transmission from NHPs to humans can occur through direct contact with bodily fluids, ingestion of contaminated resources, or through vector-mediated routes [18]. Despite this, malaria infections in wild macaques remain understudied; most research has focused on laboratory infections or single species. One comparative study demonstrated species-specific immune responses among different macaque hosts [19], emphasizing the importance of species-level investigations. Understanding these dynamics is essential for predicting zoonotic spillover and guiding control strategies. For instance, *Macaca fascicularis* (Mf) can naturally recover from infection, whereas *Macaca mulatta* (Mm) infections may be fatal, indicating species-dependent differences in susceptibility and immune response. These variations suggest that macaque species play distinct roles in parasite transmission, influencing their significance as reservoirs and vectors of human malaria.

Although significant strides have been made in understanding malaria parasites, vectors, and human host responses, research on NHPs as natural reservoirs of zoonotic *Plasmodium* remains limited. Most studies on simian malaria have focused on controlled experimental infections or individual macaque species, often neglecting interspecies variability in immune and proteomic responses. This creates a critical gap in our understanding of host–parasite dynamics under natural ecological conditions, where environmental stressors and co-evolutionary pressures may shape disease outcomes differently than in laboratory models. Previous work has highlighted the importance of species-specific immune responses; for instance, Mf can self-recover from malaria infection, whereas Mm infections can be fatal, suggesting marked differences in susceptibility and host defense strategies. A recent proteomic study of *M. arctoides* (Ma) (stump-tailed macaques) infected with malaria parasites in Thailand identified nine proteins associated with host immune responses, reinforcing the value of proteomics in uncovering biomarkers and host defense mechanisms [20]. However, this investigation was restricted to a single macaque species, leaving unresolved questions regarding whether these proteomic responses are conserved, divergent, or species-specific across other macaque hosts of zoonotic malaria. Furthermore, the extent to which these differences reflect evolutionary adaptations, ecological pressures, or parasite-specific interactions remains poorly understood.

To address these gaps, the present study undertakes a comparative proteomic analysis of three free-living macaque species, Ml, Ma, and Mf, naturally infected with zoonotic *Plasmodium* species in Thailand. By integrating high-throughput liquid chromatography-tandem mass spectrometry (LC-MS/MS) with bioinformatic approaches, this study aims to (i) identify differentially expressed serum proteins across species, (ii) characterize functional pathways and protein–protein interaction (PPI) networks involved in host immune responses, and (iii) evaluate the potential of conserved proteins as candidate biomarkers of malaria infection. Through this comparative approach, we seek to determine whether specific macaque species mount distinct or overlapping proteomic responses to infection, thereby clarifying their roles as reservoirs and models for human-relevant malaria research. Ultimately, this work provides insights into host–parasite interactions in natural populations and informs the development of surveillance tools and targeted interventions for zoonotic malaria control.

## MATERIALS AND METHODS

### Ethical approval

All experimental procedures were conducted in accordance with the Guide for the Care and Use of Laboratory Animals (National Institutes of Health, USA). Approval was obtained from the Institutional Animal Care and Use Committee of the Kasetsart University Research and Development Institute, Thailand (Protocol ID: ACKU59-SCI-011, approved July 2016). Field research was conducted under authorization from the Department of National Parks, Wildlife, and Plant Conservation, Thailand (Permit No. 0909.204/14187). All procedures were performed under veterinary supervision and adhered to ethical and professional standards for wildlife handling.

### Study period and location

Between October 2018 and January 2019, a total of 61 blood samples were collected from free-living macaques during the late rainy to winter season. These included:

- 25 Ml (14 males and 11 females) from Khao Yai National Park, Nakhon Ratchasima Province (GPS: 14.444504, 101.376237);
- 32 Ma (5 males and 7 females) from Kaeng Krachan National Park, Prachuap Khiri Khan Province (GPS: 12.240800, 99.464004);
- 4 Mf (2 males and 2 females) from Mu Ko Ranong National Park, Ranong Province (GPS: 9.838183, 98.436467).

### Macaque sampling and blood collection

Macaques were captured using baited cages (4 × 4 × 3 cm; W × L × H) with seasonal fruits and transported to temporary veterinary stations. To minimize stress, opaque cloths were used during handling. Animals were anesthetized with tiletamine-zolazepam (2–5 mg/kg) and xylazine hydrochloride (0.5–2 mg/kg). Blood samples (maximum 3 mL) were collected from the femoral vein into ethylenediaminetetraacetic acid (EDTA) tubes.

Samples were centrifuged at 2,200 × *g* for 20 min at 4°C, and plasma was preserved in liquid nitrogen before long-term storage at −80°C. Recovery was monitored by assessing respiratory rate, heart rate, and body temperature. Normal posture and mobility were restored within 60–90 min, after which macaques were released into their natural habitat. Anthropometric data (body weight, body length, limb measurements, and tail length) were recorded, alongside sex determination, dental impressions, and photographic dentition records.

### Molecular identification of Plasmodium spp.

From each EDTA blood sample, 40–50 μL was spotted onto Whatman 3MM filter paper to generate dried blood spots, later processed at the Malaria Research Center, Universiti Malaysia Sarawak. DNA was extracted using InstaGene Matrix (Bio-Rad Laboratories, USA).

Nested polymerase chain reaction (PCR) targeting small subunit ribosomal RNA was performed:

- First round (nest 1): primers rPLU1/rPLU5
- Second round (nest 2): primers rPLU3/rPLU4, with nest 1 amplicons as template.

PCR products were visualized on 2.7% agarose gels stained with SYBR Safe. Species-specific primers targeted *P. knowlesi*, *Plasmodium coatneyi*, *P. cynomolgi*, *P. inui*, and *P. fieldi* [21, 22]. This method demonstrates sensitivity of 1–6 parasites/μL of blood [23, 24]. DNA quality was verified using a NanoDrop spectrophotometer (Thermo Fisher Scientific, New York, USA) (260:280 nm ratio), and samples were stored at −20°C.

### Serum protein preparation for LC-MS/MS

Protein concentration was determined using the Lowry assay with bovine serum albumin (BSA) as a standard [25]. Five μg of serum protein was reduced with dithiothreitol (5 mM, 1 h at 60°C), alkylated with iodoacetamide (15 mM, 45 min, dark, room temperature [25°C]) and digested overnight at 37°C using trypsin (1:20 ratio). Peptides were dried, stored at −80°C, and reconstituted in 0.1% formic acid before analysis.

### Protein identification and quantification

Peptide samples were analyzed on an Ultimate3000 Nano/Capillary LC system (Thermo Scientific, UK) coupled to an HCTUltra LC-MS system (Bruker Daltonics, Germany). Separation was performed on a C18 analytical column (75 μm × 15 cm) using a gradient (5–55% acetonitrile in 0.1% formic acid) at 0.30 μL/min over 30 min. Ionization was achieved through CaptiveSpray (1.6 kV).

Spectra were acquired in positive-ion mode (m/z 150–2200) at 2 Hz. MS/MS data were searched against the UniProt Macaca database (Release 2022_04) using Mascot v2.2 [26, 27]. Stringent criteria were applied: ≤1% false discovery rate (FDR), ≥1 unique peptide, Mascot p < 0.05. Samples were analyzed in technical triplicates, and BSA standards were used for calibration.

Relative quantification employed spectral counting with *post hoc* statistical validation (p < 0.05). Blank runs were included after every 10 injections to prevent carryover.

### Statistical analysis

Total protein abundance was calculated using maximal protein expression level (PEL) values. Differentially expressed proteins (DEPs) were identified using the Kruskal–Wallis H test (p < 0.05) for three-group comparisons and Mann–Whitney U test for pairwise comparisons, applying log_2_ fold-change thresholds (±1). p-values were adjusted for FDR using the Benjamini–Hochberg method.

DEPs were mapped onto PPI networks in STRING v12.0 (https://string-db.org/) (confidence >0.900, minimum degree = 3). Functional annotation employed EuKaryotic Orthologous Groups (KOG) (https://www.hsls.pitt.edu/), Kyoto Encyclopedia of Genes and Genomes (KEGG) Orthology (KO) (https://www.genome.jp/kegg/ko.html), and GhostKOALA databases (https://www.genome.jp/kegg/pathway.html). Gene Ontology (GO) classification was performed using PANTHER v19.0 (http://pantherdb.org/) across molecular function, biological process, and cellular component domains.

**Figure 1:**
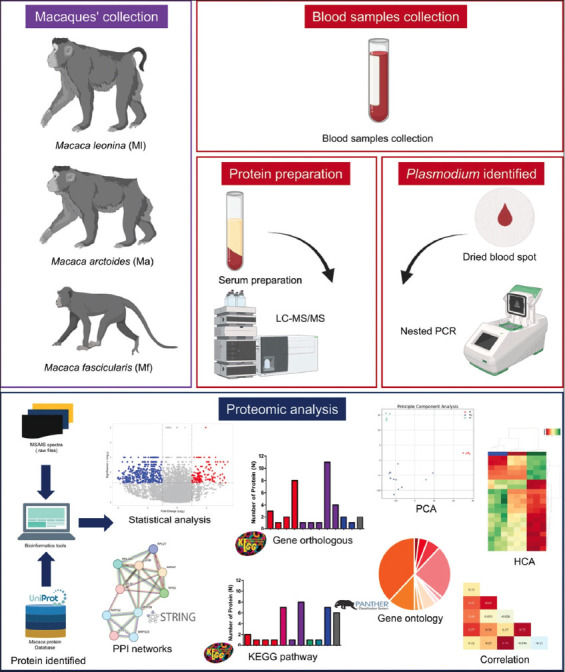
Overview of the process. The flowchart provides a comprehensive overview of the project, outlining the sample collection process to the final results.

Cluster analyses, including principal component analysis (PCA) and hierarchical heatmap clustering, were performed and visualized using Python scripts on Google Colab (https://colab.research.google.com/). Protein correlations were assessed with Spearman’s rank coefficients. Results are summarized in Figure 1.

## RESULTS

### Identification of *Plasmodium* parasites using nested PCR

All 61 macaque samples, 25 Ml, 32 Ma, and 4 Mf, were screened for *Plasmodium* infection using nested PCR from DNA extracted from dried blood spots (Table 1). Among the Ml samples, 5 of 25 (20.00%) tested positive for zoonotic malaria species, including *P. inui*, *P. cynomolgi*, and *P. fieldi*. In Ma, 16 of 32 (50.00%) were malaria-positive, with 11 (34.78%) identified as zoonotic infections involving *P. inui*, *P. cynomolgi*, *P. fieldi*, *P. coatneyi*, and *P. knowlesi*. All four Mf samples (100%) tested positive for zoonotic malaria, specifically *P. inui* and *P. fieldi*.

**Table 1:** Descriptive characteristics of macaques infected with zoonotic malaria.

Sample IDs	*Macaca* spp.	*Plasmodium* spp.
Ml01	*Macaca leonina*	Pin + Pfld
Ml02	*Macaca leonina*	Pin + Pfld
Ml03	*Macaca leonina*	Pin + Pfld
Ml04	*Macaca leonina*	Pin
Ml05	*Macaca leonina*	Pcy
Ma01	*Macaca arctoides*	Pcy + Pfld
Ma02	*Macaca arctoides*	Pcy
Ma03	*Macaca arctoides*	Pin
Ma04	*Macaca arctoides*	Pin
Ma05	*Macaca arctoides*	Pfld
Ma06	*Macaca arctoides*	Pin + Pcy + Pfld
Ma07	*Macaca arctoides*	Pfld
Ma08	*Macaca arctoides*	Pin + Pcy + Pfld
Ma09	*Macaca arctoides*	Pin + Pcy
Ma10	*Macaca arctoides*	Pfld
Ma11	*Macaca arctoides*	Pin
Mf01	*Macaca fascicularis*	Pin + Pfld
Mf02	*Macaca fascicularis*	Pin
Mf03	*Macaca fascicularis*	Pin
Mf04	*Macaca fascicularis*	Pin

Ml (n* = 5/24), Ma (n* = 11/32), and Mf (n* = 4/4). Pin = P*lasmodium inui; Pfld* = P*lasmodium fieldi; Pcy* = P*lasmodium cynomolgi; Pct* = P*lasmodium coatneyi*; Pk = P*lasmodium knowlesi.* n* = Number of macaques infected with zoonotic malaria/Number of total macaques

### Proteome identification in zoonotic malaria-infected macaques

**Figure 2:**
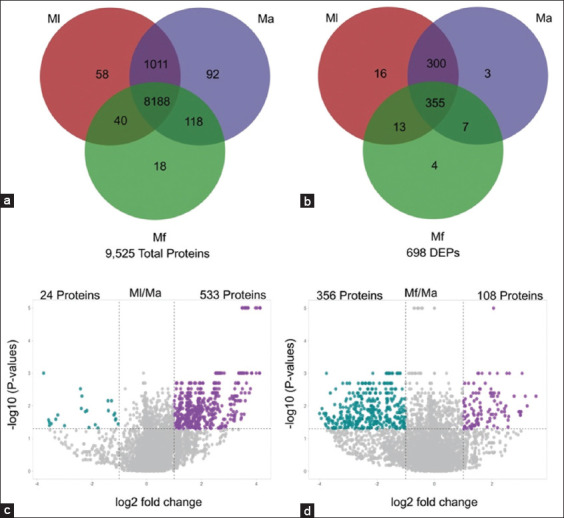
Serum protein identification in three macaques infected with zoonotic malaria. The Venn diagram represents (a) total proteins and (b) differentially expressed proteins identified in three macaques, and the volcano plot represents the number of up-/downregulated proteins expressed compared to Ma as baseline using p-values and log2 fold-change (c) Ml compared to Ma and (d) Mf compared to Ma. Ml = M. leonina, Ma = M. arctoides, and Mf = M. fascicularis. p-values were calculated using the Kruskal–Wallis test.

From the 20 macaques infected with zoonotic *Plasmodium* spp. (5 Ml, 11 Ma, 4 Mf), a total of 9,525 serum proteins were identified. Of these, 8,188 proteins were shared across all three species. Pairwise comparisons revealed 1,011 proteins unique to Ml versus Ma, 118 unique to Ma versus Mf, and 40 unique to Ml versus Mf. Species-specific unique proteins were also observed: 58 in Ml, 92 in Ma, and 18 in Mf (Figure 2a).

After applying statistical thresholds (Kruskal–Wallis test, p < 0.05; log_2_ fold change ≥ |1|), 698 DEPs were identified. These included 355 DEPs common to all species, 300 between Ml and Ma, 7 between Ma and Mf, and 13 between Ml and Mf. Species-specific DEPs included 16 unique to Ml, 3 to Ma, and 4 to Mf (Figure 2b). Notably, 577 DEPs were observed in the Ml versus Ma comparison, with 533 upregulated and 24 downregulated proteins (Figure 2c). In the Mf versus Ma comparison, 464 DEPs were identified, comprising 108 upregulated and 356 downregulated proteins (Figure 2d).

### PPI networks

**Figure 3:**
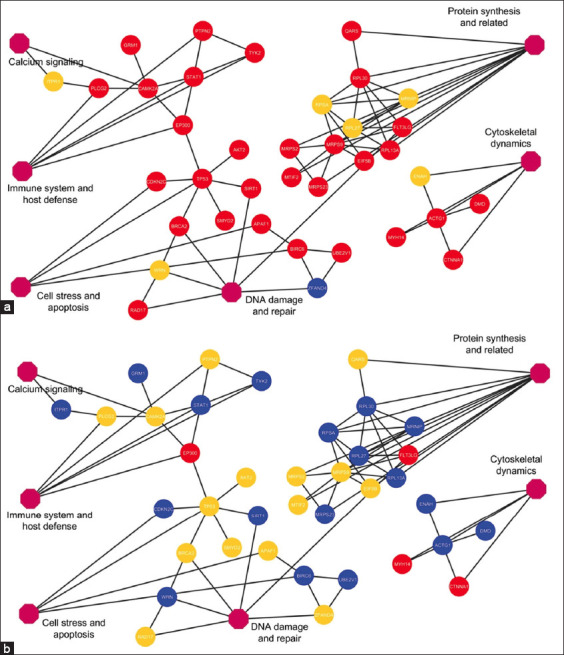
Thirty-six protein–protein interaction networks in the serum of three macaques infected with zoonotic malaria. The interaction (a) represents the fold change values of Ml to Ma, whereas the interaction (b) represents the fold change values of Mf to Ma. The node color shows the expression values, with red, blue, and orange representing upregulated, downregulated, and under-cutoff regulated proteins, respectively. Ml = *M. leonina*, Ma *= M. arctoides*, and Mf *= M. fascicularis*.

The 698 DEPs were analyzed using the STRING database, identifying 36 PPI proteins organized into four subnetworks. Expression was compared using Ma as the baseline. Of these proteins, 29 (78.38%) were upregulated in Ml, 2 (5.40%) downregulated, and 6 (16.22%) below the cutoff (Figure 3a). In the Mf versus Ma comparison, 19 proteins (51.35%) were downregulated, 3 (8.11%) upregulated, and 15 (40.54%) below cutoff expression (Figure 3b). Full details of the 36 PPIs are presented in Table 2.

**Table 2:** Thirty-six protein–protein interactions.

Gene names	Protein names	False discovery rate	Mann–Whitney U test			Mean			Ml/Ma	Mf/Ma
		
			Ml/Ma	Mf/Ma	Ml/Mf	Ml	Ma	Mf	log2FC	log2FC
Immune system and host defense mechanisms										
STAT1	Signal transducer and transcription activator	**0.031**	**0.031**	0.286	**0.047**	13.85^a^	3.03^b^	0.00^b^	2.19	−1.6
PTPN2	Tyrosine protein phosphatase non-receptor type	**0.032**	**0.018**	0.527	**0.021**	16.19^a^	6.91^b^	11.23^b^	1.23	0.7
PLCG2	1-Phosphatidylinositol 4,5-bisphosphate phosphodiesterase gamma	**0.046**	**0.007**	0.756	**0.021**	18.23^a^	9.04^b^	12.75^b^	1.01	0.5
TYK2	Tyrosine protein kinase	**0.032**	0.092	0.202	**0.014**	16.45^a^	4.65^a,b^	0.00^b^	1.82	−2.22
EP300	Histone acetyltransferase	0.037	**0.001**	0.083	0.166	10.97^a^	0.00^b^	4.25^a,b^	3.46	2.09
Cell stress and apoptosis										
APAF1	Apoptotic Peptidase-Activating Factor 1	**0.041**	**0.043**	0.443	**0.038**	15.44^a^	5.60^b^	3.83^b^	1.46	−0.55
TP53	Cellular tumor antigen p53 expression	**0.030**	**0.031**	0.626	**0.038**	14.97^a^	5.01^b^	3.55^b^	1.58	−0.50
CDKN2C	Cyclin-dependent 2C kinase inhibitor	**0.028**	**0.027**	0.061	**0.014**	14.19^a^	7.03^b^	0.00^b^	1.01	−2.81
BIRC6	Baculoviral IAP repeat-containing 6	**0.030**	0.070	0.202	**0.014**	16.89^a^	4.57^a,b^	0.00^b^	1.88	−2.19
DNA damage and repair										
MRNIP	MRN complex-interacting protein	**0.043**	0.225	**0.029**	**0.020**	16.07^a^	15.74^a^	7.61^b^	0.03	−1.05
RAD17	RAD17 Checkpoint Clamp Loader Component	**0.030**	**0.038**	0.768	**0.038**	17.28^a^	4.27^b^	3.78^b^	2.02	−0.17
SIRT1	Deacetylase sirtuin-type domain-containing protein	**0.026**	**0.007**	0.202	**0.014**	17.92^a^	4.75^b^	0.00^b^	1.92	−2.25
WRN	DNA helicase	**0.032**	0.065	0.174	**0.018**	18.57^a^	9.60^a,b^	4.14^b^	0.95	−1.21
BRCA2	The tower domain-containing protein	**0.041**	**0.082**	0.937	0.166	11.88^a^	2.62^b^	3.66^a,b^	2.18	0.48
ZFAND4	Ubiquitin-like domain-containing protein	**0.045**	0.061	0.622	**0.014**	0.00^a^	11.62^a,b^	16.86^b^	−3.54	0.54
Protein synthesis and related processes										
QARS	Glutaminyl-tRNA synthetase	**0.027**	**0.033**	0.937	**0.038**	15.94^a^	2.83^b^	3.82^b^	2.50	0.43
MRPS2	Mitochondrial ribosomal protein S2 expression	**0.032**	**0.023**	0.894	0.081	16.05^a^	5.57^b^	7.20^a,b^	1.53	0.37
MRPS23	MRP-S23 domain-containing protein	**0.030**	0.061	0.202	**0.047**	14.88^a^	4.31^a, b^	0.00^b^	1.79	−2.11
MRPS9	Mitochondrial ribosomal S9 protein	**0.050**	**0.012**	0.399	**0.014**	16.34^a^	1.59^b^	0.00^b^	3.36	−0.67
MTIF2	Tr-type G domain-containing protein	**0.044**	**0.052**	1.000	0.237	15.57^a^	4.14^b^	4.21^a,b^	1.91	0.02
UBE2V1	Ubiquitin-conjugating E2 variant 1	**0.027**	**0.023**	0.286	**0.014**	16.88^a^	3.15^b^	0.00^b^	2.42	−1.66
EIF5B	Eukaryotic translation initiation factor 5B expression	**0.049**	0.339	0.399	0.131	9.93^a^	1.63^a^	0.00^a^	2.61	−0.70
RPL13A	60S ribosomal L13a protein	**0.041**	0.171	0.094	**0.014**	18.55^a^	8.38^a,b^	0.00^b^	1.15	−3.07
RPL27	60S ribosomal L27 protein	**0.037**	0.052	**0.039**	**0.020**	18.90^a^	15.76^a^	7.79^b^	0.26	−1.02
RPL30	60S ribosomal L30 protein	**0.032**	0.129	**0.140**	0.014	17.29^a^	6.02^a,b^	0.00^b^	1.52	−2.59
RPSA	Small ribosomal subunit uS2	**0.047**	0.255	0.094	**0.047**	14.63^a^	7.96^a,b^	0.00^b^	0.88	−2.99
Calcium signaling										
CAMK2A	Calcium/calmodulin-dependent protein kinase	**0.028**	**0.018**	0.423	**0.038**	16.82^a^	6.88^b^	3.92^b^	1.29	−0.81
ITPR1	Inositol 1,4,5-trisphosphate receptor type 1	**0.031**	0.467	**0.007**	**0.014**	17.06^a^	15.03^a^	0.00^b^	0.18	−3.91
Cytoskeletal dynamics										
ACTG1	Actin, cytoplasmic 2	**0.025**	**0.028**	0.202	**0.014**	18.09^a^	4.95^b^	0.00^b^	1.87	−2.31
CTNNA1	Catenin alpha 1	**0.026**	**0.016**	**0.031**	0.564	16.17^a^	2.69^b^	12.67^a^	2.59	2.23
ENAH	WH1 domain-containing protein	**0.030**	0.068	0.071	**0.018**	17.83^a^	12.11^a,b^	4.10^b^	0.56	−1.56
MYH14	Myosin heavy chain 14	**0.032**	**0.011**	0.331	0.248	17.50^a^	6.19^b^	12.87^a,b^	1.50	1.06
Other proteins										
DMD	Dystrophin	**0.025**	**0.011**	0.286	**0.014**	17.47^a^	3.16^b^	0.00^b^	2.47	−1.66
GRM1	Glutamate receptor, Metabotropic 1	**0.047**	0.534	0.094	**0.014**	14.10^a^	6.59^a,b^	0.00^b^	1.10	−2.72
SMYD2	SMYD2 N-lysine methyltransferase	**0.047**	**0.046**	0.897	0.245	18.21^a^	7.76^b^	8.96^a, b^	1.23	0.21
AKT2*	AKT serine/threonine kinase 2	**0.028**	**0.002**	0.564	**0.014**	13.20^a^	1.54^b^	0.00^b^	3.09	−0.63

* Proteins identified in previous studies.

^a,b^Significant with Mann–Whitney U test at p < 0.050. Bold p values = significant with false discovery rate and Mann–Whitney U test at p < 0.050. Signal transducer and activator of transcription

### Functional annotation and pathway analysis

Functional annotation of the 36 PPI proteins was conducted using KOG and KO orthologous databases:

#### KOG classification

**Figure 4:**
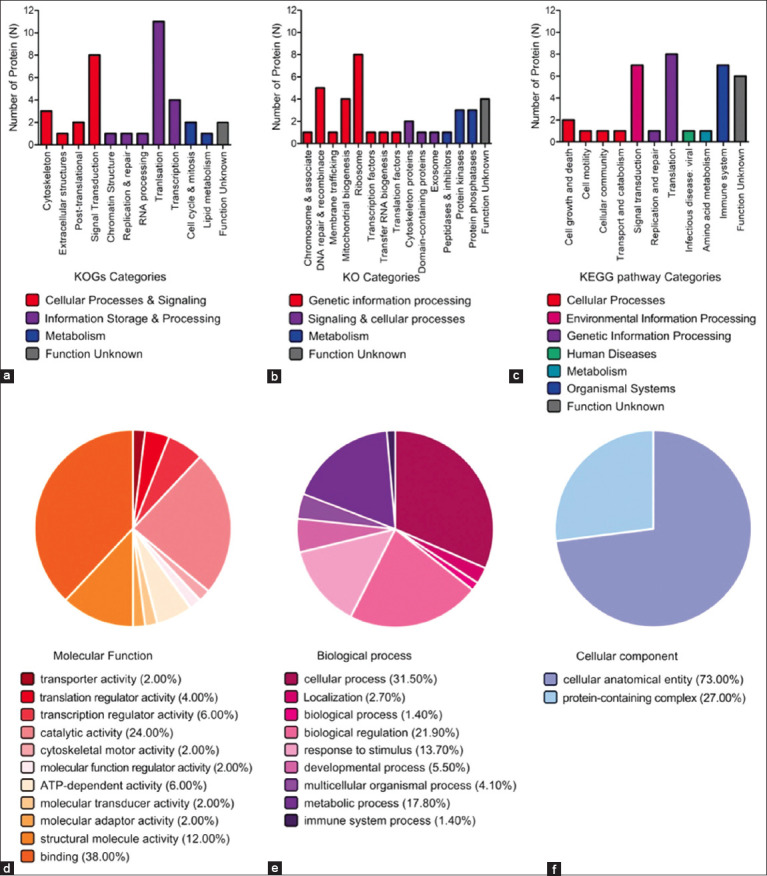
Functional enrichment of 36 protein–protein interaction networks. Gene orthologs were used to categorize proteins using the (a) EuKaryotic Orthologous Groups and (b) Kyoto Encyclopedia of Genes and Genomes (KEGG) Orthology databases. (c) The pathway was described using the Kyoto Encyclopedia of Genes and Genomes pathway database. Gene ontology was used to classify protein function into three categories: (d) molecular function, (e) biological process, and (f) cellular component.

- 14 proteins in *Cellular Processes and Signaling* (57.14% signal transduction, 21.43% cytoskeletal organization, 14.29% post-translational modification, 7.14% extracellular structure)
- 18 proteins in *Information Storage and Processing* (44.44% translation, 22.22% transcription, 11.11% chromatin structure/replication/repair/RNA processing)
- 3 proteins in *Metabolism* (66.67% cell cycle and mitosis, 33.33% lipid metabolism)
- 2 proteins unclassified (Figure 4a).

#### KO classification

- 24 proteins in *Genetic Information Processing* (33.33% ribosomal function, 29.17% DNA repair/recombination, 16.67% mitochondrial biogenesis, others in chromosome-associated proteins, membrane trafficking, transcription factors, transfer RNA (tRNA) biogenesis, translation factors)
- 4 proteins in *Cellular Processes and Signaling* (50.00% cytoskeletal functions, 25.00% domain-containing proteins, 25.00% exosomal components)
- 7 proteins in *Metabolism* (42.86% protein kinases, 42.86% phosphatases, 14.28% peptidases/inhibitors)
- 4 proteins unclassified (Figure 4b).

#### KEGG pathway enrichment

- 5 proteins in *Cellular Processes* (40.00% cell growth/death, 20.00% each in motility, cellular community, transport/catabolism)
- 7 proteins in *Environmental Information Processing* (signal transduction)
- 1 protein in *Human Diseases* (viral infection).
- 1 protein in *Metabolism* (amino acid metabolism)
- 7 proteins in *Organismal Systems* (immune system)
- 9 proteins in *Genetic Information Processing* (88.89% translation, 11.11% replication/repair)
- 6 proteins unclassified (Figure 4c).

#### GO analysis (PANTHER)

- *Molecular function*: 38% binding, 24% catalytic activity, 12% structural molecule, 6% transcription regulation, 6% ATP-dependent activity (Figure 4d)
- *Biological processes*: 31.50% cellular processes, 21.90% biological regulation, 17.80% metabolism, 13.70% stimulus response, 5.50% development (Figure 4e)
- *Cellular components*: 73% cellular anatomical entities, 27% protein-containing complexes (Figure 4f).

### Cluster analysis

**Figure 5:**
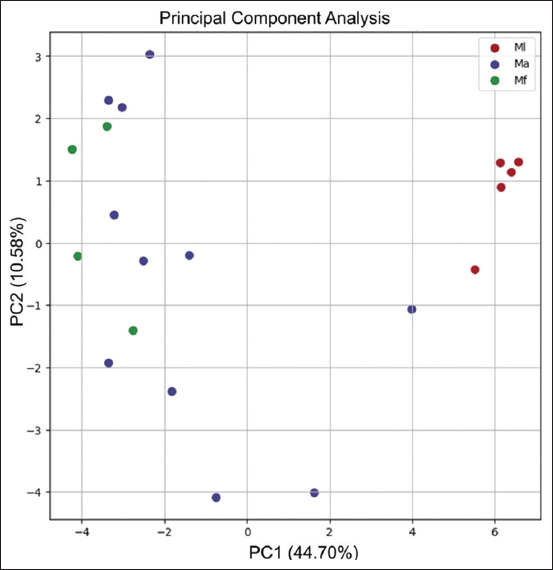
Principal component (PC) analysis of 36 proteins from three macaque species. The PCs collectively explained 55.28% of the total variance, with PC1 accounting for 44.70% and PC2 contributing to 10.58%. The red, blue, and green dots represent the three macaque species, and the 20 macaques included *M. leonina* (Ml), *M. arctoides* (Ma), and *M. fascicularis* (Mf), respectively.

**Figure 6:**
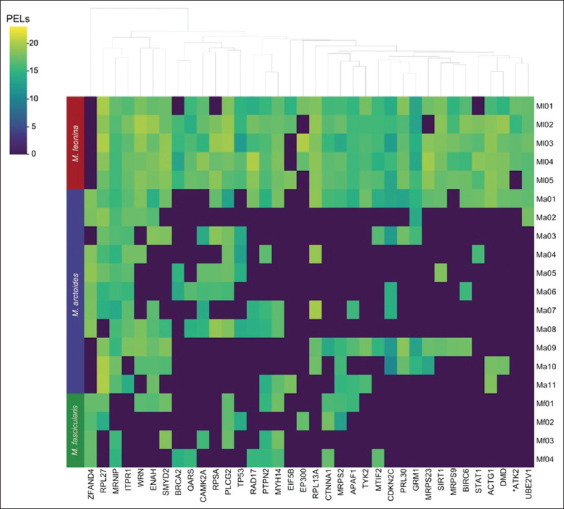
Clustering heatmap analysis of the 36 proteins. The vertical bands in red, blue, and green represent the macaque samples *Macaca leonina* (Ml), *Macaca arctoides* (Ma), and *Macaca fascicularis* (Mf), respectively. The X-axis shows the names of the 36 proteins. The color gradient of the heatmap reflects the protein expression levels.

PCA using the 36 PPI proteins explained 55.28% cumulative variance (PC1: 44.70%; PC2: 10.58%). PCA revealed distinct separation of Ml samples, while Ma and Mf overlapped (Figure 5). Hierarchical clustering (HCA) showed high expression of most proteins in Ml, with lower expression in Ma and Mf, except ubiquitin-like domain-containing protein (ZFAND4). Without root fixation, Ma and Mf displayed mixed clustering patterns, consistent with PCA (Figure 6).

### Key malaria-associated proteins

Nine proteins previously associated with malaria [20] were reanalyzed across species (Table 3). Six proteins, CARD domain-containing protein, interleukin 1 receptor-associated kinase 1, nicotinamide phosphoribosyltransferase, non-specific serine/threonine protein kinase (RAF1), BRHD domain-containing protein, and sterol regulatory element-binding transcription factor, showed no significant interspecies differences. In contrast, mothers against a decapentaplegic homolog and SWI/SNF complex subunit SMARCC2 isoform were significantly differentially expressed, being upregulated in Ml and downregulated in Mf relative to Ma. AKT serine/threonine kinase 2 emerged as the central hub in the 36-node PPI network and was notably upregulated in Ml.

**Table 3:** Nine important proteins from previous studies.

Gene name	Protein name	False discovery rate	Log2 fold change	

			Ml/Ma	Mf/Ma
SMAD7	Mothers against a decapentaplegic homolog	**0.021**	1.95	−1.89
SMARCC2	SWI/SNF complex subunit SMARCC2 isoform c	**0.025**	3.23	3.09
AKT2	AKT serine/threonine kinase 2	**0.028**	3.09	−0.63
RELB	BRHD domain-containing protein	0.095	−0.76	−3.06
IRAK1	Interleukin 1 receptor-associated kinase 1	0.098	0.72	−0.37
NAMPT	Nicotinamide phosphoribosyltransferase	0.165	0.89	0.72
SREBF1	Sterol regulatory element-binding transcription factor	0.169	−1.87	−0.07
RAF1	Non-specific serine/threonine protein kinase	0.214	0.22	−1.67
CARD10	CARD domain-containing protein	0.718	−0.49	−0.27

Bold p values = significant with false discovery rate at p* <* 0.050. Ml = M*. leonina*, Ma = M*. arctoides,* and Mf* = M. fascicularis*, SMAD7 = Mothers against a decapentaplegic homolog, SMARCC2 = SWI/SNF complex subunit SMARCC2 isoform c, AKT2 = AKT serine/threonine kinase 2, RELB = BRHD domain-containing protein, IRAK1 = Interleukin 1 receptor associated kinase 1, NAMPT = Nicotinamide phosphoribosyltransferase, SREBF1 = Sterol regulatory element-binding transcription factor, RAF1 = Non-specific serine/threonine protein kinase, CARD10 = CARD domain-containing protein, SWI/SNF = Switch/sucrose non-fermentable, RHD = Rel homology domain.

## DISCUSSION

To inform surveillance and intervention strategies and advance efforts toward the global elimination of malaria by 2030, extensive research has been conducted on drug resistance, parasite biology, mosquito vector distribution, and human host responses. These efforts have improved our understanding of the mechanisms that have enabled malaria control in many regions. However, zoonotic malaria presents a substantial barrier to its eradication. Limited research has been conducted on infections in non-human primates. In this study, we investigated serum protein expression in three macaque species infected with zoonotic malaria parasites using a proteomics-based approach integrated with bioinformatic analyses. This study aimed to identify host-specific immune signatures and inform strategies for surveillance and intervention in zoonotic malaria transmission.

### Immune system and host defense mechanisms

Among the 36 proteins identified, five were associated with immune responses: Signal transducer and activator of transcription 1 (STAT1), Tyrosine-Protein Phosphatase Non-Receptor Type 2 (PTPN2), phospholipase C Gamma 2 (PLCG2), E1A binding protein P300 (EP300), and Tyrosine Kinase 2 (TYK2). In our study, three proteins belonging to the immune system and host defense categories were significantly upregulated in Ml, while the other two groups (Ma and Mf) were downregulated. STAT1 is a key mediator of interferon (IFN) signaling through the Janus Kinase (JAK)-STAT pathway. It becomes phosphorylated and activated by type I (IFNα, IFNβ, IFNε, IFNκ, IFNω), type II (IFNγ), and type III (IFNλ) IFNs, initiating early antiparasitic defense responses [28, 29]. STAT1 also promotes the expression of proinflammatory cytokines, such as TNF-α and IL-6, and induces apoptosis [30]. Tyrosine-Protein Phosphatase Non-Receptor Type 2 negatively regulates the JAK-STAT pathway by dephosphorylating [31] key components, such as JAK1/3 and STAT1/3, thereby inhibiting their activation and nuclear translocation. Reduced PTPN2 expression in macrophages enhances JAK-STAT signaling, thereby amplifying immune responses [32]. PTPN2 helps to mitigate tissue damage associated with hyperinflammation during infection by preventing excessive immune activation [33]. PLCG2 is a key enzyme in the phosphatidylinositol signaling pathway, playing a crucial role in calcium signaling, cytokine production, and the activation of immune cells. It plays an essential role in signal transduction through B-cell and T-cell receptors (BCR and TCR), both of which are crucial for adaptive immunity against parasites [34, 35]. The simultaneous upregulation of these proteins in Ml suggests a coordinated immune response, wherein innate immunity (mediated by STAT1) and adaptive immunity (through PLCG) were activated, while regulatory mechanisms (PTPN2) were concurrently engaged to mitigate excessive immune activation. This expression profile indicates a robust yet tightly regulated immune defense against infection. Nonetheless, the temporal dynamics of the infection, whether representing an early or late stage, remain unclear and warrant further investigation.

In addition, we identified TYK2, a protein that was upregulated in the Ml, downregulated in the Mf, and regulated between the Ml and the Mf. It is a member of the JAK family and is a critical component of cytokine signaling, primarily through the JAK-STAT pathway [29]. This pathway is essential for interferon-, interleukins-, and over 50 other cytokines-mediated signal transduction [36]. This suggests heightened cytokine signaling through the JAK-STAT pathway, which amplifies responses to interferons and interleukins in the Ml. Finally, EP300 was identified as upregulated in the Ml, downregulated in the Ma, and regulated between the Ml and the Ma. It is a histone acetyltransferase that modulates the expression of immune-related genes, including those involved in inflammatory responses and phagocyte function, such as the formation of neutrophil extracellular traps [37]. It regulates STAT1 acetylation in coordination with histone deacetylases, thereby modulating nuclear factor kappa B activity and apoptosis [38]. However, all five proteins classified under the immune system and host defense categories were also present in humans. Further investigations are warranted to evaluate their expression profiles in humans and to determine whether these patterns were comparable to, or distinct from, those observed in other macaque species, which could provide valuable insights for selecting appropriate models for studies on the immune system and host defense.

### Cell stress and apoptosis

Apoptosis, a form of programmed cell death, is initiated when cells incur irreparable damage from pathogens or internal stress, such as DNA damage. This mechanism prevents the spread of infection by eliminating compromised cells, which are subsequently cleared by immune cells [39]. The three proteins were upregulated in the Ml, while the Ma and Mf were downregulated. Apoptotic Protease Activating Factor 1 (APAF1) is a central regulator of the intrinsic apoptotic pathway. Upon binding to cytochrome c, APAF1 assembles the apoptosome complex, which activates caspases [40]. Tumor Protein P53 (TP53) promotes apoptosis in parasite-infected cells, thereby restricting parasite replication and dissemination. In immune cells, TP53 also regulates responses to infection-induced stress and promotes type I IFN responses [41, 42]. Cyclin-dependent kinase inhibitor 2C (CDKN2C) enforces cell cycle arrest in response to infection-related stress. This pause in proliferation enables immune cells to prioritize effector functions, such as pathogen clearance, over replication, thereby enhancing immune efficacy [43, 44].

However, one protein was identified as significantly upregulated in the Ml, while Mf was downregulated (Ma between Ml and Mf). Baculoviral IAP Repeat Containing 6 (BIRC6) is an IAP inhibitor that blocks caspase activation, thereby preventing cell death. While this function supports the survival of immune cells, it may also enable parasite-infected cells to evade apoptosis [45]. This expression profile indicates a tightly regulated balance between cellular apoptosis and survival. The upregulation of APAF1, TP53, and CDKN2C facilitates pathogen elimination by enhancing apoptosis and optimizing immune responses, whereas BIRC6 protects against excessive cell death, thereby preserving immune homeostasis during infection.

### DNA damage and repair

Host cells often undergo stress and release cytokines and signaling molecules during infection by bacteria, fungi, viruses, or protozoan parasites, which triggers apoptosis or activates immune defenses, thereby limiting pathogen spread. However, excessive production of reactive oxygen species (ROS) can cause severe DNA damage. If this damage exceeds the repair capacity, apoptosis is triggered [46]. *Plasmodium* infection induces high levels of oxidative stress and ROS production, resulting in DNA damage in host immune and endothelial cells [47, 48]. Several proteins safeguard genomic stability and cellular function under these conditions. We identified six proteins involved in DNA damage and repair and found that three were upregulated only in the Ml. MRN complex-interacting protein (MRNIP) interacts with the MRN complex (MRE11–RAD50–NBS1), a critical sensor and mediator of DNA double-strand break (DSB) repair. MRNIP facilitates the proper assembly and function of the MRN complex, promoting efficient DNA damage repair, particularly in immune and endothelial cells under infection-induced stress [49, 50]. RAD17 Checkpoint Clamp Loader Component (RAD17) is a central regulator of DNA damage response (DDR) and ATR-mediated signaling. It promotes the recognition and repair of DNA lesions induced by replication stress and enforces cell cycle arrest, thereby preventing genomic instability transmission [51]. Sirtuin 1 (SIRT1), a NAD^+^-dependent deacetylase, enhances DNA repair pathways by deacetylating core DDR proteins. It also modulates immune cell metabolism and inflammatory signaling, thereby balancing immune activity to limit collateral tissue damage [52, 53]. The observed upregulation of MRNIP, RAD17, and SIRT1 in the Ml group indicates an active DDR alongside mechanisms that preserve genomic integrity and immune equilibrium during infection. This expression pattern suggests that the host orchestrates a robust DDR to maintain genomic stability while concurrently regulating metabolic and inflammatory pathways to support effective yet controlled immune responses.

WRN (Werner Syndrome RecQ Helicase-Like Protein) was upregulated in the Ml, downregulated in the Mf, and Ma (between the Ml and the Mf). It is a member of the RecQ helicase family and is essential for DNA replication, repair, and telomere maintenance. It preserves genome stability by resolving aberrant DNA structures, preventing replication fork collapse, and ensuring DNA integrity during immune activation [54, 55]. Breast Cancer Type 2 Susceptibility Protein was upregulated in Ml, downregulated in Ma, and Mf (between Ml and Ma). It plays a pivotal role in the repair of homologous recombination-mediated DNA. It recruits RAD51 to DSB sites, facilitating accurate DNA repair and maintaining genomic integrity in immune and endothelial cells that are under stress [56, 57]. Interestingly, only one of 36 proteins was downregulated in the Ml group. ZFAND4 contributes to protein quality control and cellular stress responses during DNA repair. In immune cells, it supports cellular resilience against oxidative damage and inflammation, helping to maintain homeostasis during infection [58, 59]. The downregulation of ZFAND4 in the Ml, along with the upregulation of other stress- and immune-response proteins, indicates a partial attenuation of cellular stress defenses. This suggests that although immune activation and DNA repair processes were active, protein homeostasis may be impaired during infection, potentially impacting overall cell viability.

The Ml exhibits a coordinated response to infection, characterized by upregulated DNA repair and immune-regulatory proteins that maintain genomic stability and immune balance, while a slight reduction (ZFAND4) in protein stress defenses indicates mildly compromised protein homeostasis.

### Protein synthesis and related processes

During infection, protein synthesis is critically regulated because host cells must rapidly produce immune effectors while restricting the synthesis of proteins co-opted by pathogens for replication or nutrient acquisition [60, 61]. Among the 36 proteins examined, 11 were involved in protein synthesis. Five of these, 60S ribosomal L13a protein (RPL13A), 60S ribosomal L27 protein (RPL27), 60S ribosomal L30 protein (RPL30), small ribosomal subunit uS2 (RPSA), and eukaryotic translation initiation factor 5B (EIF5B), are associated with cytosolic ribosomes. RPL13A, RPL27, and RPL30 are core constituents of the large ribosomal subunit (60S) [62, 63] and are essential for the translation of immune mediators such as cytokines, signaling proteins, and transcription factors. RPSA, a component of the small ribosomal subunit (40S), is pivotal for ribosome assembly and translation initiation, supporting protein biosynthesis under immune stress [63, 64]. EIF5B plays a central role in assembling the translation initiation complex, enabling efficient protein synthesis initiation [65]. Four additional proteins, mitochondrial ribosomal protein S2 (MRPS2), MRP-S23 domain-containing protein (MRPS23), mitochondrial ribosomal S9 protein (MRPS9), and mitochondrial translation initiation factor 2 (MTIF2), are mitochondrial ribosomal components. MRPS2, MRPS23, and MRPS9 are structural elements of the mitochondrial ribosome, which govern intramitochondrial protein synthesis, which is essential for energy production during immune activation [66]. MTIF2 initiates mitochondrial translation by assembling the translation initiation complex, thereby supporting mitochondrial function under stress [67]. Glutaminyl-tRNA synthetase (QARS) catalyzes the aminoacylation of tRNA, a crucial step in maintaining translational fidelity and efficiency [68]. The upregulation of these synthetic proteins in the Ml group during malaria suggests enhanced translational activity, enabling the generation of immunomodulatory proteins and sustaining mitochondrial energy production, thereby supporting an effective immune response and cellular resilience.

### Calcium signaling

Calcium ions are pivotal second messengers in immune activation induced by *Plasmodium* infection. Dynamic changes in intracellular calcium levels initiate signaling cascades that modulate immune cell function [69, 70]. Calcium/Calmodulin-Dependent Protein Kinase II Alpha (CAMK2A) was upregulated only in Ml. It transduces calcium signals to downstream effectors, regulating immune responses in T lymphocytes and endothelial cells [71, 72]. Inositol 1,4,5-Trisphosphate Receptor Type 1 (ITPR1) was upregulated in the Ml and Ma, whereas Mf was downregulated. This protein mediates the release of calcium from the endoplasmic reticulum, orchestrating cellular events such as gene expression and inflammatory activation [73, 74]. The upregulation of CAMK2A and ITPR1 in Ml during malaria reflects an active calcium-mediated immune response that promotes T cell activation, gene transcription, and inflammatory signaling to support effective host defense.

### Cytoskeletal dynamics

Cytoskeletal dynamics profoundly influence host defense, tissue remodeling, immune cell trafficking, and pathogen clearance. Disruptions in these processes can compromise immune surveillance and facilitate parasite invasion, thereby affecting disease progression [75, 76]. Actin Gamma 1, Catenin Alpha 1, Enabled Homolog, and Myosin Heavy Chain 14 coordinately regulate actin filament organization, cell adhesion, and motility. These proteins are essential for maintaining vascular integrity, facilitating the recruitment of immune cells, and supporting structural adaptation in response to *Plasmodium* infection [77]. These proteins were upregulated more in the Ml group, while some Ma and Mf were similarly expressed and some were downregulated. Its upregulation during malaria infection in Ml groups may indicate that the host actively mobilizes the cytoskeleton in response to the parasite.

### Other proteins

We identified four proteins as upregulated in the Ml groups. Dystrophin, a key structural protein in muscle cells, anchors the extracellular matrix to the actin cytoskeleton and modulates cellular inflammation. During *Plasmodium* infection, which induces systemic inflammation, dystrophin may influence inflammatory responses within muscle tissue [78]. Glutamate Metabotropic Receptor 1, which is primarily involved in neuronal glutamate signaling, also contributes to neuroinflammatory processes central to the pathogenesis of cerebral malaria by regulating brain-specific inflammatory responses [79]. SET and MYND Domain Containing 2 (SMYD2), a histone methyltransferase, mediates epigenetic regulation through the methylation of histones and transcription factors, thereby modulating gene expression profiles associated with immune function, cellular differentiation, and proliferation during infection [80].

### Nine important proteins

A previous study by Ruengket *et al*. [20] reported that nine proteins play critical roles in host defense mechanisms through the modulation of lipid metabolism, induction of apoptosis, activation of cytokine responses, type I interferon signaling, and pro-inflammatory pathways across Ma with mono-malaria, multiple-malaria, and non-infected conditions. In the current study, six of nine selected proteins were classified as non-significant. This suggests that their expression is consistently altered upon malaria infection, irrespective of macaque species, thereby indicating their potential as universal biomarkers of infection across Ml, Ma, and Mf. Therefore, these proteins were suitable for further study in humans to develop them into biomarker proteins.

A comprehensive analysis of 45 proteins (36 identified through PPI networks and nine previously reported immune-relevant proteins) using PELs, PCA, HCA, log_2_ fold change, and statistical significance testing revealed species-specific expression patterns. Most proteins were upregulated in Ml, whereas Ma and Mf were predominantly downregulated, with several proteins exhibiting similar expression levels across species. These expression trends may reflect evolutionary divergence and distinct ecological exposures to Plasmodium, suggesting that co-evolutionary pressures may drive species-specific adaptive responses to malaria infection. However, species-specific protein regulation may represent adaptive immune mechanisms shaped by prolonged evolutionary exposure to *Plasmodium* infection. These findings characterize protein expression patterns across macaque species during infection with malaria. Integrating these data with human studies could help identify primate expression profiles most comparable to humans, thereby guiding the selection of optimal models for future malaria research.

## CONCLUSION

This study provides novel insights into the proteomic responses of three free-living macaque species, Ml, Ma, and Mf, naturally infected with zoonotic *Plasmodium* spp. By integrating serum proteomics with bioinformatics, we identified 9,525 serum proteins, of which 698 were differentially expressed, and 36 proteins formed robust PPI networks. Ml exhibited predominant upregulation of immune, apoptotic, DNA repair, protein synthesis, calcium signaling, and cytoskeletal proteins, whereas Ma and Mf showed downregulation trends. Importantly, nine malaria-associated proteins previously reported by Ruengket *et al*. [20] were reanalyzed, and six demonstrated consistent expression across species, underscoring their potential as universal biomarkers of zoonotic malaria.

The identification of conserved immune-related proteins such as STAT1, PLCG2, PTPN2, EP300, and TYK2 highlights their translational potential for developing diagnostic markers and improving surveillance tools. The upregulation of DNA repair and apoptosis regulators in Ml indicates that certain macaque species mount more robust protective responses, suggesting that species-specific host responses must be considered in zoonotic malaria risk assessments. These findings can guide the selection of optimal primate models for malaria research, facilitating translational studies relevant to human infections.

A major strength of this work lies in its field-based design, using wild macaques under natural ecological conditions rather than controlled laboratory models. This approach captures realistic host–parasite interactions shaped by evolutionary and ecological pressures, thus improving the relevance of findings for zoonotic transmission scenarios. The combined use of proteomics, bioinformatics, and pathway analyses enhances confidence in the robustness of the identified proteins and pathways. However, the study is limited by the relatively small sample size, particularly for Mf (n = 4), which may reduce statistical power. Additionally, environmental, dietary, and seasonal factors may influence proteomic profiles, and the cross-sectional design limits temporal interpretations of infection stages.

Future research should expand to include larger sample sizes across multiple geographic regions and employ longitudinal monitoring of macaque populations to capture the dynamic proteomic responses during different stages of infection. Comparative analyses with human malaria patients will be crucial for validating macaque proteins as biomarkers and for identifying the most suitable macaque models for human-relevant malaria studies. Integrating multi-omics approaches (genomics, transcriptomics, and metabolomics) could provide a systems-level understanding of host–parasite interactions.

In conclusion, free-living macaques exhibit species-specific proteomic responses to zoonotic *Plasmodium* infections, with Ml showing stronger and more coordinated immune activation compared with Ma and Mf. The identification of conserved and differentially regulated proteins advances our understanding of host–parasite co-evolution and provides a foundation for biomarker discovery and surveillance strategies. These findings underscore the importance of NHPs in malaria research and highlight the need to integrate wildlife monitoring into global malaria elimination programs.

## DATA AVAILABILITY

All raw data are publicly available on the jPOST repository under accession numbers JPST004052 (https://repository.jpostdb.org/preview/203373860468b60e8458642, access key 5188) and PXD067961.

## AUTHORS’ CONTRIBUTIONS

PR: Performed the experiment, analyzed the data, and wrote the manuscript. SR: Performed the metaproteomic analysis and wrote the manuscript. DT: Identified the locations and collected blood samples. CU: Undertook the molecular detection of the malaria parasite and data analysis. WF: Conceived, designed, and supervised the study and revised the manuscript. All authors have read and approved the final version of the manuscript.
